# How Vision Affects Kinematic Properties of Pantomimed Prehension Movements

**DOI:** 10.3389/fpsyg.2013.00044

**Published:** 2013-02-07

**Authors:** Takao Fukui, Toshio Inui

**Affiliations:** ^1^Department of Intelligence Science and Technology, Graduate School of Informatics, Kyoto UniversityKyoto, Japan

**Keywords:** reach-to-grasp movement, pantomimed action, vision, dorsal and ventral streams, grip configuration

## Abstract

When performing the reach-to-grasp movement, fingers open wider than the size of a target object and then stop opening. The recorded peak grip aperture (PGA) is significantly larger when this action is performed without vision during the movement than with vision, presumably due to an error margin that is retained in order to avoid collision with the object. People can also pretend this action based on an internal target representation (i.e., pantomimed prehension), and previous studies have shown that kinematic differences exist between natural and pantomimed prehension. These differences are regarded as a reflection of variations in information processing in the brain through the dorsal and ventral streams. Pantomimed action is thought to be mediated by the ventral stream. This implies that visual information during the movement, which is essential to the dorsal stream, has little effect on the kinematic properties of pantomimed prehension. We investigated whether an online view of the external world affects pantomimed grasping, and more specifically, whether the dorsal stream is involved in its execution. Participants gazed at a target object and were then subjected to a 3-s visual occlusion, during which time the experimenter removed the object. The participants were then required to pretend to make a reach-to-grasp action toward the location where the object had been presented. Two visual conditions (full vision and no vision) were imposed during the pantomimed action by manipulating shutter goggles. The PGA showed significant differences between the two visual conditions, whereas no significant difference was noted for terminal grip aperture, which was recorded at the movement end. This suggests the involvement of the dorsal stream in pantomimed action and implies that pantomimed prehension is a good probe for revealing the mechanism of interaction between the ventral and dorsal streams, which is also linked to embodied cognition.

## Introduction

People perform adaptive motor behaviors in their daily lives, and these adaptive behaviors are assumed to emerge from continuous interaction among the nervous system, body, and environment (Chiel and Beer, 1997). Embodied cognition argues that the sensorimotor process mediating this adaptive control of bodies in environments is tightly related to the cognitive system (e.g., Clark, 1997; Wilson, 2002). In particular, the hand developed in a remarkably human-specific manner and the actions by this body part added variety to human lives and led ultimately to civilization. The old mot “The hand is the window on to the mind,” which is ascribed to Immanuel Kant (cf. Tallis, 2004), also indicates that the hand serves as a substantial interface between the external world and the individual self.

Reaching for and grasping an object is one of the basic functions of the human hand in daily life. Following Jeannerod’s (1981, 1984) pioneering studies, this fundamental human skill has been a research focus for the last three decades (e.g., Castiello and Begliomini, 2008; Grafton, 2010; Rosenbaum et al., 2012 for recent reviews). The reach-to-grasp movement consists of two components: a transport component, which is thought to direct the arm to the spatial location of the target, and a manipulation component, which is involved in grasping a three-dimensional object (Jeannerod, 1981, 1984). Jeannerod (1981) was the first to systematically describe the behavioral aspects of the grasping action in which the fingers first open gradually to form the appropriate configuration for the target object to be grasped (“preshaping”). The fingers then continue to open wider than the size of the target object and stop opening at a point about 60–70% into the movement (i.e., the peak grip aperture, PGA), after which they enclose the object, finally touching its surface (e.g., Jeannerod and Marteniuk, 1992). Accomplishing this movement requires appropriate visuomotor transformation, which indicates that visual information is essential for the online control of goal-directed movements. When visual information from the entire visual field is absent during prehension, this invokes a significantly larger PGA (Wing et al., 1986; Jakobson and Goodale, 1991; Bradshaw and Elliott, 2003; Fukui and Inui, 2006). This is due to the greater margin of hand aperture, which allows for error in movement and prevents collision of the fingers with the target object (e.g., Wing et al., 1986). Therefore, PGA has been regarded as an indicator of the influence of online vision on grasping.

In addition to these types of goal-directed movements, motor behavior can be performed toward an object, even when that object is no longer present, based on the memory of the object by imagining its properties (i.e., pantomimed action). Despite our assumption that people could pantomime well and replicate the motor performance of (natural/real) goal-directed movements, previous studies have demonstrated that the kinematics of pantomimed prehension differ quite substantially from prehension to an existing object (e.g., Goodale et al., 1994). Specifically, these researchers found that, when compared to normal prehension, pantomimed prehension consistently reached lower peak velocities, tended to last longer, followed more curvilinear trajectories, and undershot the target location. PGA was also smaller when pantomiming than when grasping the existing objects. Unlike normal prehension, pantomimed prehension has no haptic feedback due to lack of a target object; thus people have to configure their terminal grip aperture (TGA) according to a memory representation of the target object. When the participants could see a visual image of the target object via a mirror apparatus while they reached for it, a significant difference in the TGA was noted between with and without haptic feedback conditions (Bingham et al., 2007). These researchers also found that mixing the trials with and without haptic feedback in one experimental session resulted in an appropriate configuration of the TGA in the no feedback condition, indicating the importance of haptic calibration opportunities (see also Schenk, 2012).

In association with cognitive and sensorimotor processes, two relatively parallel streams have been proposed to explain visual information processing in the brain; namely the ventral stream, which projects from the primary visual cortex to the inferotemporal cortex, and the dorsal stream, which projects from the primary visual cortex to the posterior parietal cortex (Ungerleider and Mishkin, 1982). The ventral stream was initially proposed to play a critical role in the identification and recognition of objects (“what” pathway), whereas the dorsal stream was thought responsible for localizing those objects in space (“where” pathway). However, research revealed that “where” did not fully express the functions of dorsal streams. For example, some patients with damage to the posterior parietal cortex (i.e., the dorsal stream) were found unable to orient the hand and form an appropriate grasp, in addition to the inability to reach a proper spatial location (e.g., Rondot et al., 1977; Perenin and Vighetto, 1988; Jakobson et al., 1991). Therefore, Goodale and Milner (1992) focused on the differences in the output systems served by each stream. Specifically, they proposed that the ventral stream plays a major role in constructing a perceptual representation of the visual world and the objects within it, while the dorsal stream mediates the visual control of actions directed at those objects (the “How” pathway; Goodale, 2011).

The observed differences in kinematics between pantomimed and natural motor behaviors suggest that different control is exerted on pantomimed actions from that of natural goal-directed motor behavior. Specifically, pantomimed motor behavior might be guided by the ventral system, whereas natural goal-directed motor behavior is mediated by the dorsal stream (Westwood et al., 2000; Milner and Goodale, 2006). This argument was strengthened by a neuropsychological study that investigated an optic ataxic patient who suffered with visuomotor difficulties due to severe bilateral damage to the posterior parietal lobes (Milner et al., 2001). The PGA of this patient’s pantomimed prehension scaled according to the object size, implying that visual memory for this action was appropriately used and that the intact ventral stream could contribute to this motor behavior.

Although the contribution of the ventral stream to pantomimed prehension was revealed by these previous studies, the nature of the involvement of the dorsal stream with execution of the pantomimed prehension remains unclear. The primal function of the dorsal stream is the online transformation of visual information into action execution (Jeannerod et al., 1995; Desmurget et al., 1999; Pisella et al., 2000; Grea et al., 2002). The question becomes whether visual information of the environment affects the performance of pantomimed prehension, particularly the configuration of the grip aperture, such as the PGA and the TGA. There are two possibilities each for the kinematics of PGA and TGA when manipulating vision during movement.

Concerning the PGA,

1. In pantomimed movement, there is no risk of collision with an object, so participants do not have to account for a margin of error. Therefore, no difference would be expected in the PGA between the full vision and no vision conditions during pantomimed movement.
2. If online information extraction of the external world (assumed to mediated by the dorsal stream), in addition to an internal representation of the target object, contributes to the appropriate configuration of grip aperture, a significant difference in the PGA between the full vision and no vision conditions could emerge.

Concerning the TGA,

1. TGA could have more precise scaling in the vision than the no vision conditions due to the benefits of view from hand and/or spatial references (e.g., location of an experimental table), in addition to an internal representation of the target object.
2. An internal representation of the target object is a dominant and primary factor for determining the TGA, so no difference would be expected in the TGA between the full vision and no vision conditions.

In this study, we investigated whether visual information during movement affects the kinematics of pantomimed prehension; specifically, we determined which of the above mentioned possibilities would be more plausible, by manipulating the vision with crystal shutter goggles during movement execution.

## Materials and Methods

### Participants

Seven self-reported right-handed students (mean: 24.4 years of age, SD: 2.70; one female) participated in the experiment. All participants reported normal or corrected-to-normal vision and none of them had any motor or sensory abnormalities. They were naive with regard to the purpose of the experiment, and gave their informed consent according to the Declaration of Helsinki.

### Apparatus and stimulus

**Figure 1:**
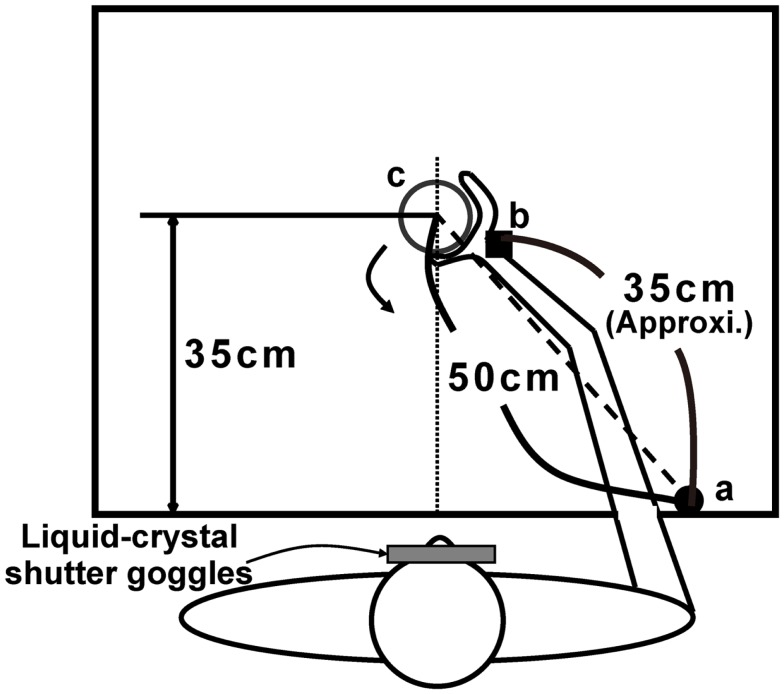
**Table layout for the experiment**. a: Initial hand position on the pressure-sensitive switch; b: position of cubical receiver on the wrist when grasping; c: position of target presentation. The reaching distance recorded by the receiver was approximately 35 cm from the starting position.

Participants were equipped with liquid crystal shutter goggles (Takei Scientific Instruments Co., Ltd., Niigata, Japan) and seated comfortably on a chair in front of a table (120 cm × 75 cm) in a room with natural lighting. As illustrated in Figure 1, a target object was presented along the participant’s sagittal plane, with a distance between the target object and the starting position of 50 cm. A pressure-sensitive switch was located at the starting position, which was approximately in line with the participant’s right shoulder. Three wooden cylinders (4, 6, and 9 cm in diameter and 11 cm in height) were used as target objects. An electromagnetic motion tracking sensor FASTRAK system (Polhemus, Colchester, VT, USA) was used for measuring the position of wrist (the head of ulna), while the aperture between the thumb and middle finger was calculated by a data glove (Virtual Technologies, Inc., Palo Alto, CA, USA). The temporal resolution of the motion tracking sensor was 120 Hz and that of the data glove was 100 Hz. The liquid crystal shutter goggles take about 3 ms to become transparent and about 20 ms to become opaque. A workstation Octane (SGI Japan, Ltd., Tokyo, Japan) controlled the apparatus and recorded the kinematics.

### Procedure

Participants were told to place their right hand at the starting position before each trial and to begin each trial with the tips of the thumb and middle finger touching each other. Goggles were opaque before trials. The experiment consisted of two sessions: prehension to a real object and pantomimed prehension. Participants first performed prehension to a real object (“natural” prehension) with vision, and then they performed pantomimed prehension with or without vision, presented in a random fashion.

A natural grasping task with vision was performed as a baseline condition. We assumed that an experience of real interaction with a target object via natural prehension is a prerequisite for an appropriate pantomimed action; therefore, the pantomime task was preceded by the natural grasping task. In the real grasping task, participants were required to reach for and grasp the presented target object and then lift and move the object 5–10 cm toward their bodies, under full vision (cf. Fukui and Inui, 2006; and, see also Figure 1). We did not test natural prehension without vision because our main interest was determining how the visual information of the environment during movement execution influences the configuration of pantomimed prehension movements. We were concerned that the control modulated by the visual context (i.e., full vision or no vision) in natural prehension session would influence the control of the following pantomimed prehension.

Participants performed pantomimed prehension movements as follows: first, goggles became transparent following a beep signal and stayed transparent for 1 s. During this period, the participants were required to memorize the target properties (i.e., size, location, etc.). After this period, the goggles became opaque and remained in this condition for 3 s (i.e., delay). During this time, the experimenter removed the target object. Two viewing conditions were designed for the subsequent procedure: (i) Pantomimed action with vision (PV), where the goggles again became transparent after a beep and participants performed a pantomimed action to the memorized target object; (ii) Pantomimed action with no vision (PNV), where the goggles remained opaque and the participants performed the pantomimed action, cued by the beep, according to the memorized target object. The two viewing conditions (PV/PNV) and three target sizes (4, 6, and 9 cm) were presented randomly, with nine trials for each combination (i.e., a total of 54 experimental trials). In addition to the pantomimed movement of grasping, the participants were required to pretend to lift and move the object 5–10 cm toward their bodies.

In the real grasping (RV) session, nine trials for each object size (4, 6, and 9 cm) were presented randomly (i.e., a total of 27 experimental trials). A 3-s delay was also inserted in this session, as well as in the pantomimed session, and the target object was not removed during this delay (cf. Milner et al., 2001). Before both pantomime and real grasping sessions, participants were required to perform each action a few times as practice trials (within five trials) according to the instructions.

### Data processing and analysis

The initiation of the movement was defined as the time that the participant’s hand released from the pressure-sensitive switch. The termination of movement was defined as the time point at which the maximal value of the distance between the wrist and the starting point (i.e., the point in which direction of the wrist movement was changed) was recorded (Zaal and Bootsma, 1993; Bootsma et al., 1994; Fukui and Inui, 2006).

The positional data given by Cartesian coordinates in three dimensions from the receiver were recorded and filtered offline by a second-order dual-pass Butterworth filter with a cut-off frequency of 10 Hz. Further offline analysis included computation of wrist velocity from the filtered position signal. We also calculated two grasp component values; specifically, PGA and TGA (the aperture between thumb and middle finger at the point in time when the changes in grasp configuration were stable). As an index of movement variability (reach distance, PGA, and TGA), standard deviations across trials were computed for each participant.

The mean data for each dependent variable were analyzed with an ANOVA, with object size (4, 6, and 9 cm) and the task (PV, PNV, and RV) as within-participant factors (alpha level = 0.05). Huynh–Feldt adjustments to the degrees of freedom were performed when necessary. As described earlier, participants did not perform real prehension without vision because previous experience of both full and no vision conditions during natural prehension was expected to influence online control in the subsequent pantomimed prehension session. That is why we incorporated PV, PNV, and RV into one within-participant factor as a task. Our interest is the comparisons of each dependent variable between PV and PNV conditions and those between PV and RV conditions. As *post hoc* comparisons, we performed paired *t*-tests, using the Bonferroni correction, on the mean values for PV and PNV conditions, for PV and RV conditions, and for each size.

## Results

We found lower peak wrist velocity and smaller PGA (except for the 9-cm object) in the pantomimed prehension tasks when compared to natural prehension tasks, as demonstrated by Goodale et al. (1994). In addition to these results, kinematic differences were found in pantomimed prehension between the full vision and no vision conditions. Specifically, we found a larger PGA when pantomiming with no vision than when pantomiming with full vision. At the same time, no significant difference was noted for the TGA values between the full vision and no vision conditions.

### Reach distance and reach distance variability

**Figure 2:**
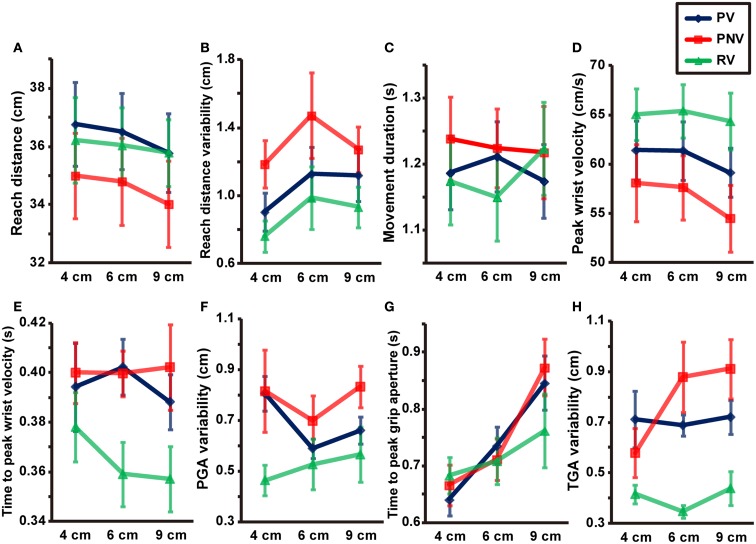
**Mean values of kinematic parameters in each condition**. Reach distance **(A)**, reach distance variability **(B)**, movement duration **(C)**, peak wrist velocity **(D)**, time to peak wrist velocity **(E)**, PGA variability **(F)**, time to peak grip aperture **(G)**, and TGA variability **(H)**. PV, PNV, and RV indicate pantomime prehension with vision, pantomime prehension without vision, and real prehension with vision, respectively. Error bars indicate the standard errors of the values between participants.

Reach distance (Figure 2A) showed a main effect of task [*F*(2, 12) = 4.550, *p* = 0.034, partial η^2^ = 0.431], but no significant main effect of size [*F*(2, 12) = 3.261, *p* = 0.074] and no interactions between the two factors [*F*(4, 24) = 0.825, *p* = 0.522]. Further analysis revealed a significant difference between PV and PNV conditions (*p* < 0.001), but no significant difference between PV and RV conditions (*p* = 0.297). The reach distance was undershot when visual information was not available during pantomimed prehension, whereas the distance was comparable to that of natural prehension when visual information was available.

Reach distance variability (Figure 2B) showed a main effect of task [*F*(2, 12) = 7.288, *p* = 0.009, partial η^2^ = 0.548], but no significant main effect of size [*F*(2, 12) = 2.890, *p* = 0.095] and no interaction between the two factors [*F*(4, 24) = 0.316, *p* = 0.865]. Further analysis revealed a significant difference between PV and PNV conditions (*p* = 0.012), indicating that the lack of available visual information during pantomime action increased in the reach distance variability.

### Movement duration

A significant interaction between size and task [*F*(2.990, 17.942) = 3.375, *p* = 0.041, partial η^2^ = 0.360] was evident, although no main effects were noted [size: *F*(2, 12) = 0.350, *p* = 0.712, task: *F*(2, 12) = 1.318, *p* = 0.304]. We found a simple main effect of size in the RV condition (*p* = 0.007), but further analysis revealed no significant differences among the different sizes (Figure 2C).

### Transport component

#### Peak wrist velocity

Both size [*F*(2, 12) = 13.905, *p* < 0.001, partial η^2^ = 0.699] and task [*F*(2, 12) = 13.546, *p* < 0.001, partial η^2^ = 0.693] had significant effects on the peak wrist velocity (Figure 2D). No significant interaction was noted between size and task [*F*(4, 24) = 1.217, *p* = 0.330]. Further analysis revealed significant differences between the PV and RV conditions (*p* < 0.001), indicating that pantomimed action was slower than natural prehension. We also found a significant difference between the PV and the PNV conditions (*p* < 0.001), suggesting further reduction of the velocity was observed when visual information was not available during pantomimed prehension. We also found significant differences between the 4 and 9-cm objects (*p* = 0.001) and between the 6 and 9-cm objects (*p* < 0.001). This result might be due to the grip manner of the 9-cm object in which the fingers were almost completely extended to their capacities to ensure that the object was stably held, leading to a cautious action manner even in the pantomimed conditions.

#### Time to peak wrist velocity

Time to peak velocity (Figure 2E) showed a main effect of task [*F*(2, 12) = 5.719, *p* = 0.018, partial η^2^ = 0.488], but no significant main effect of size [*F*(2, 12) = 1.174, *p* = 0.342] and no interaction between the two factors [*F*(4, 24) = 0.804, *p* = 0.535]. Further analysis revealed a significant difference between the PV and RV conditions (*p* < 0.001), indicating a later timing to peak wrist velocity in pantomimed prehension than in real grasping, under full vision condition.

### Manipulation component

#### Peak grip aperture and variability of PGA

**Figure 3:**
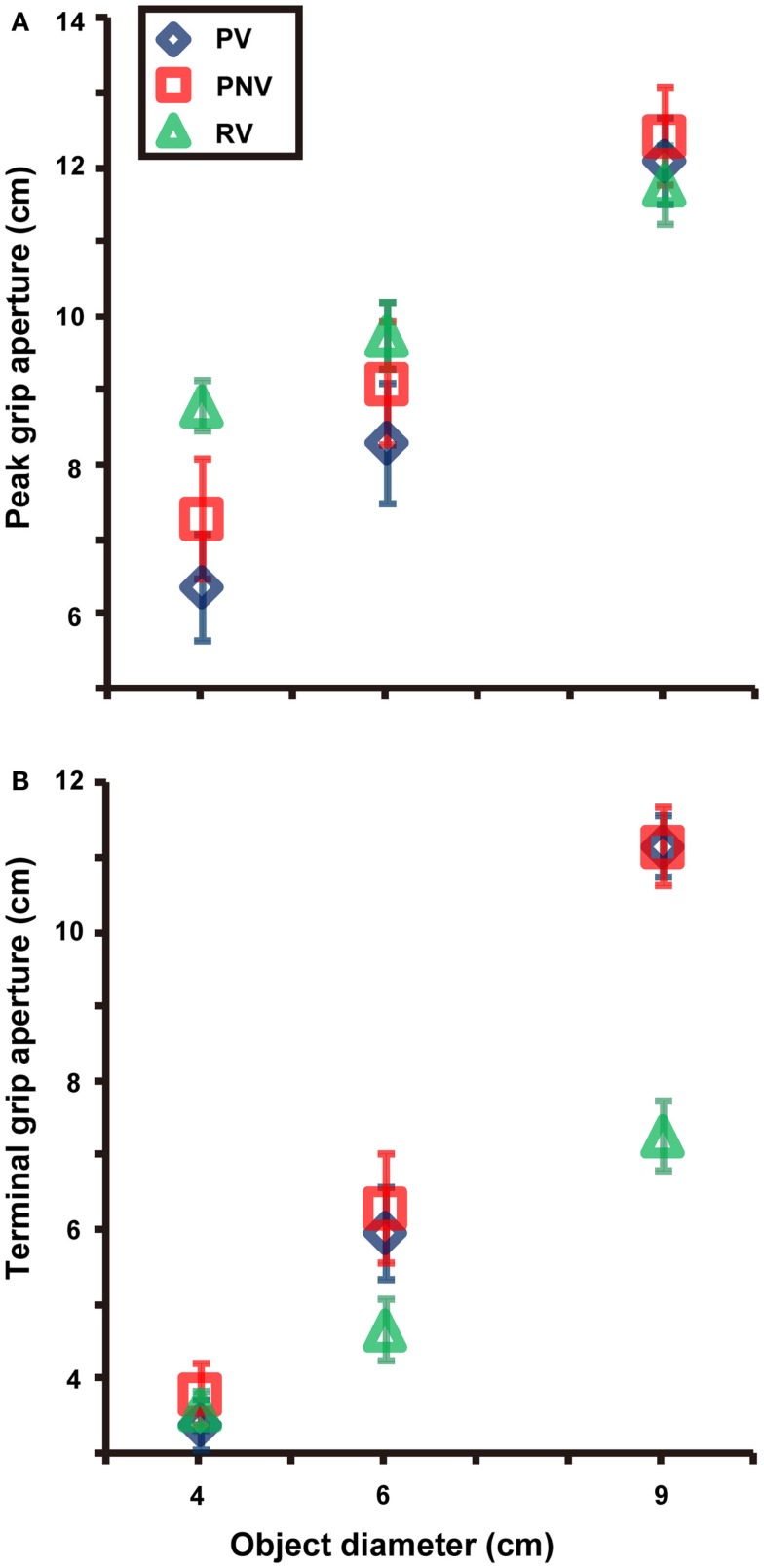
**Mean values of peak grip aperture (A) and terminal grip aperture (B) in each condition**. Differences between PV and PNV conditions for the 4 and 6-cm objects were found, whereas no difference of terminal grip aperture was noted between the PV and PNV conditions for any object size. PV, PNV, and RV indicate pantomime prehension with vision, pantomime prehension without vision, and real prehension with vision, respectively. Error bars indicate the standard errors of the values between participants.

Both size [*F*(2, 12) = 130.140, *p* < 0.001, partial η^2^ = 0.956] and task [*F*(2, 12) = 4.341, *p* = 0.038, partial η^2^ = 0.420] significantly affected the PGA. We found a significant interaction between size and task [*F*(4, 24) = 26.797, *p* < 0.001, partial η^2^ = 0.817]. We also found significant differences between PV and PNV (*p* = 0.003) and between PV and RV (*p* = 0.008) for the 4-cm object and a significant difference between PV and PNV (*p* = 0.006) for the 6-cm object (see Figure 3A). In other words, the PGA was significantly larger when visual information was not available in the pantomimed action. The results imply that visual information appeared to affect the configuration of grip aperture in pantomimed prehension, although no difference was found for the 9-cm object, presumably due to a kind of ceiling effect constrained by the hand structure. The variability of PGA (Figure 2F) showed a main effect of task [*F*(2, 12) = 6.088, *p* = 0.015, partial η^2^ = 0.504], but no main effect of size [*F*(2, 12) = 0.991, *p* = 0.400] or interaction between size and task [*F*(4, 24) = 0.826, *p* = 0.522]. Further analysis revealed a significant difference between the PV and RV conditions (*p* = 0.017), but the difference between the PV and PNV conditions was not significant (*p* = 0.400). This result suggests that this value would be modulated depending on the existence of the target object during movement.

#### Time to peak grip aperture

Time to PGA (Figure 2G) showed a main effect of size [*F*(2, 12) = 19.125, *p* < 0.001, partial η^2^ = 0.761], and a significant interaction between the size and task [*F*(4, 24) = 4.945, *p* = 0.005, partial η^2^ = 0.452], but no main effect of task [*F*(2, 12) = 1.008, *p* = 0.394]. Further analysis revealed a significant difference between the 4 and 9-cm objects (*p* < 0.001) in the PV condition, as well as significant differences between the 4 and 9-cm objects (*p* = 0.005) and between the 6 and 9-cm objects (*p* = 0.002) in the PNV condition. No significant differences among target sizes were noted in the RV condition (*p* = 0.074). The time to PGA in pantomimed prehension tasks would shift later according to the increase of the object size.

#### Terminal grip aperture and variability of TGA

Both size [*F*(2, 12) = 198.662, *p* < 0.001, partial η^2^ = 0.971] and task [*F*(2, 12) = 17.319, *p* < 0.001, partial η^2^ = 0.743] significantly affected TGA. A significant interaction was noted between size and task [*F*(4, 24) = 48.496, *p* < 0.001, partial η^2^ = 0.890]. Further analysis revealed a significant difference between the PV and RV conditions for the 9-cm object (*p* < 0.001). No significant difference was noted between the PV and the PNV conditions for any object size. This suggested that the availability of visual information during pantomimed prehension did not affect the TGA (see Figure 3B). The variability of TGA (Figure 2H) showed a main effect of task [*F*(2, 12) = 32.616, *p* < 0.001, partial η^2^ = 0.845] but no main effect of size [*F*(2, 12) = 1.261, *p* = 0.318] and no interaction between size and task [*F*(1.857, 11.140) = 1.488, *p* = 0.266]. Further analysis revealed a significant difference between the PV and RV conditions (*p* < 0.001), suggesting larger variability in pantomimed movements, under full vision condition.

## Discussion

The current study explored: (i) whether kinematic differences exist between pantomimed prehension and natural grasping, as shown previously (e.g., Goodale et al., 1994); and (ii) whether visual information during movement affects the kinematics of pantomimed prehension. We confirmed the kinematic differences between natural and pantomimed prehension movements with full vision, as Goodale et al. (1994) had previously demonstrated. Specifically, when participants performed pantomimed action, they showed lower peak wrist velocity and smaller PGA (except for the 9-cm object) when compared to the natural prehension task, although no significant difference in the reach distance was noted in the current experiment.

At the same time, the kinematic differences were found in pantomimed prehension between the full vision and no vision conditions. Specifically, we found an undershot reach distance and a larger variability, a lower peak wrist velocity, and larger PGA when pantomiming with no vision than when pantomiming with full vision, which suggested that a view of the external environment affects the execution of pantomimed prehension. Previous studies in a goal-directed movement (e.g., Watt et al., 2000) demonstrated that a situation of visual uncertainty induced an undershot bias, and the current findings of the transport component (i.e., the undershot reach distance and its larger variability with a lower peak velocity when pantomiming with no vision) suggest that this visual uncertainty also influences the pantomimed action in a similar manner of a goal-directed action.

The interesting finding in our study was the significant difference observed in the PGA between the full and no vision conditions, while no significant difference was observed in the TGA. As for the larger PGA without vision, we could not ascribe this result to a decay of the memory for performing this action, as Hesse and Franz (2009) pointed out in their natural prehension experiments. That is because, in contrast to natural grasping, even if the target representation is decayed and more vague in the no vision condition than in the full vision condition, physical contact does not need to occur with an object in a pantomimed action, so the PGA does not need to increase in the no vision condition as there is no need for an error margin to avoid a collision with the object, as described in the Introduction. Furthermore, TGA, which is configured according to this representation, would have also showed the difference between these two conditions, but we did not find such difference in the TGA (and its variability). Rather, the target representation, which was assumed to be reflected in TGA, showed a stable property that was immune to the decay. This result implies that an internal representation about the target object for the pantomiming might not be influenced by the availability of visual information. In fact, the TGA for the 4 and 6-cm objects corresponded to the object size (although, the 9-cm object was somehow overestimated), which implied that the pantomime grip aperture might depict the form of the object (Laimgruber et al., 2005). The interpretation for the smaller PGA obtained in the full vision condition is that environmental visual information and/or view of the hand contributed to a “better” grip aperture configuration even in the pantomimed action (i.e., memory-guided movements; cf. Ietswaart et al., 2001; Heath, 2005). This “better” configuration does not mean that the pantomimed prehension with vision shows more similar kinematic properties to natural grasping than that without vision; rather, it implies that in the full vision condition, there is no additional opening of grip aperture when pantomimed prehension is performed. In addition to the online information extraction, another interpretation of the current results is that participants, presumably implicitly, would try to “simulate” the grip configuration of the real grasping action in the task of pantomimed prehension. Specifically, in spite of the requirement of performing the pantomimed prehension, participants modulate the grip aperture by taking into account the environmental (visual) context (i.e., full vision or no vision). Note that, as described in the introduction, a larger PGA without vision was observed in the real grasping. Although such modulation in the pantomimed prehension would not be necessary because there is no real object to be grasped, the modulation of the PGA, according to the visual context, would suggest involvement of the body in the cognition of the external world (cf. Witt and Proffitt, 2008).

The pantomimed prehension seen in the current study could be characterized as a motor behavior into which a delay period is inserted between the target object presentation and action phases. Milner and Goodale (2006) proposed that a delay between stimulus presentation and grasping led to a shift from dorsal to ventral control of the movement because the dorsal stream does not retain a visual memory for more than 2 s; therefore, a memory-guided action introduced by a delayed period is mediated by the ventral stream (see also Westwood and Goodale, 2003). However, Himmelbach and Karnath (2005) found that the movement error of a pointing task performed by patients with optic ataxia decreased linearly with longer delays and argued that residual dorsal processing still exists in delayed movements and that there is a gradual change between the dorsal and ventral streams. The current results (i.e., the significant difference in the PGA according to the availability of visual information as opposed to no significant difference in the TGA) suggest that the TGA is generated by the perceptual representation of the visual world, which is immune to the availability of visual information and is mediated by the ventral stream, while the PGA difference reflects an online information extraction mediated by the dorsal one. Therefore, generating the grip configuration of the delayed pantomimed prehension would be contributed by both the ventral and dorsal streams, along with the findings from the delayed pointing task by Himmelbach and Karnath (2005) (see also Franz et al., 2009; Hesse and Franz, 2009; Janczyk et al., 2010 for arguing the “one representation” hypothesis).

As for the related neural basis of this study, an fMRI study by Króliczak et al. (2007) found that pantomimed grasping invokes activation primarily in several areas in the right posterior parietal lobe (e.g., the superior parietal lobe and posterior parts of the intra-parietal sulcus) as well as in some other areas (e.g., the area overlapping both the right medial temporal gyrus and the superior temporal sulcus) in the right hemisphere. Recently, Makuuchi et al. (2012) demonstrated that execution of pantomimed prehension requires the interaction between dorsal and ventral streams. Specifically, they found significant intrinsic connections between the anterior intraparietal sulcus (AIP, dorsal) and posterior inferior temporal gyrus (pITG, ventral), consistent with the anatomical connection between these areas (Borra et al., 2008). These fMRI studies indicate that the dorsal stream is involved in execution of pantomimed action; this supports our current finding for the PGA, which would result from the contribution of the dorsal stream. Rizzolatti and Matelli (2003) proposed that the dorsal visual stream could be functionally subdivided into (i) the dorso-dorsal pathway running from V6 to V6a and a medial intra-parietal region (MIP) in the superior parietal lobule (SPL), functioning in the online control of action; and (ii) the ventro-dorsal pathway running from the medial superior temporal (MST) area to the inferior parietal lobule (IPL), functioning in motor control, action understanding, and space perception (see also Pisella et al., 2006). The open question remaining for further investigation is which dorsal stream (dorso-dorsal or ventro-dorsal pathways) is dominantly involved with the current PGA result. Specifically, the question is what function (motor aspect and/or a kind of space perception) is reflected on the PGA result observed in the present study (cf. Neggers et al., 2006; Schenk, 2006; Cavina-Pratesi et al., 2011).

Pantomime research traditionally focuses on tool-use pantomime actions (e.g., Goldenberg, 2009) while only a few studies have investigated reach-to-grasp pantomimed action. Recently, Binkofski and Buxbaum (in press) proposed that the dorso-dorsal system was characterized as the “grasp” system for the purposes of reach-to-grasp actions and that the ventro-dorsal stream was characterized as the “use” system for the specific skilled actions associated with familiar objects. In addition to real prehension studies, further research on reach-to-grasp pantomimed action is essential to clarify the mechanism of the “grasp” system. In summary, the results presented here indicate that pantomimed action is mediated by the coordination of the ventral and dorsal streams. These observations suggest that this action might be a good probe for revealing the mechanism of interaction between the ventral and dorsal streams (cf. Rossetti et al., 2000; Cloutman, in press).

## Conflict of Interest Statement

The authors declare that the research was conducted in the absence of any commercial or financial relationships that could be construed as a potential conflict of interest.
